# Mental Health Status Among Perimenopausal Women in Urban Slums: A Cross-Sectional Study

**DOI:** 10.7759/cureus.74578

**Published:** 2024-11-27

**Authors:** Sudhakar Sundaramoorthy, Pradeep TS

**Affiliations:** 1 Community Medicine, Sri Devaraj Urs Medical College, Sri Devaraj Urs Academy of Higher Education and Research, Kolar, IND

**Keywords:** anxiety, depression, perimenopausal women, stress, urban slum

## Abstract

Background

Menstruation is linked to psychological issues, particularly during its cessation. The premenopausal, perimenopausal, and postmenopausal stages of a woman’s life are associated with a higher likelihood of mental health concerns. This study aims to assess the prevalence of stress, anxiety, and depression, as well as identify factors associated with these conditions among perimenopausal women living in urban slums.

Material and methods

This cross-sectional study was conducted over the course of one year among urban slum women in their perimenopausal age, who were identified through a preliminary survey. The DASS-21 scale questionnaire was used to assess their mental health status. To examine the association between various factors and stress, anxiety, and depression, the Chi-square test was applied, with a significance level set at a p-value less than 0.05. Additionally, binary logistic regression analysis was performed to determine the OR.

Results

A total of 148 perimenopausal urban slum women participated in the study, of whom 89 (60.1%) were homemakers, 85 (57.4%) had completed primary schooling, and 63 (42.6%) had comorbidities. The mean age of the participants was 43.6 years, and the mean age of menarche was 12.4 years. Regarding mental health, 124 women (83.8%) experienced moderate stress, 112 women (75.7%) had moderate anxiety, and 82 women (55.4%) reported severe depression. Age and marital status were significantly associated with depression, while educational status was notably associated with stress.

Conclusions

The present study demonstrated a higher prevalence of stress, anxiety, and depression among perimenopausal women, with factors such as educational status, marital status, and age being significantly associated with their mental health. It is essential to raise awareness about the commonality of mental health issues during the perimenopausal stage and provide strategies to help women cope with these challenges.

## Introduction

Menstruation is a natural physiological process in women, but it can sometimes lead to health challenges. While there are specific symptoms associated with the cessation of menstrual cycles, many women experience psychological issues during this time [1]. Research has shown that menopausal women often face a reduced quality of life due to symptoms such as night sweats and vaginal dryness, which can affect physical health, self-esteem, and mental well-being [2]. The menopausal transition and postmenopausal phases are critical periods where women are more susceptible to various health-related issues, including metabolic syndrome due to insulin resistance and altered mental health [3]. Menopause is defined by the permanent cessation of menstruation for 12 consecutive months, at which point a woman is considered postmenopausal. The perimenopausal period, which precedes the final menstrual period (FMP), is often associated with a range of symptoms [4].

India currently has approximately 43 million women experiencing menopause, a number that is expected to double by 2026, reaching around 103 million. As the proportion of premenopausal women continues to rise, the proportion of postmenopausal women will also increase, highlighting the need for the healthcare sector to address the health challenges faced by this growing demographic [5]. Hormonal fluctuations during perimenopause are associated with various physical and psychological symptoms. Depression and anxiety-related disorders are leading causes of years lost due to premature mortality or disability in developed countries. Studies have shown that irregular menstrual cycles are linked to higher levels of depressive symptoms and perceived stress [6]. A population-based study in Korea found that menstrual cycle irregularity could impair mental health and sleep hygiene [7]. Additionally, women with preexisting mental health conditions may experience changes in their symptoms and response to treatment during perimenopause and postmenopause, potentially leading to poorer long-term physical health outcomes [8].

Raising awareness about health issues during menopause can significantly impact how women perceive and manage their health during the perimenopausal phase. One of the most common psychiatric morbidities during this time is depression and anxiety, which often go unnoticed [9]. A cross-sectional study in India among perimenopausal and menopausal women found that 21.9% experienced moderate anxiety, and 24.76% had clinical depression [10]. Another study conducted in Indian urban slums reported a depression rate as high as 55%. While there is substantial research on mental health in menopausal women, fewer studies focus on the perimenopausal group [11]. Against this background, our study aims to assess the prevalence of stress, anxiety, and depression among perimenopausal urban slum women, as well as to identify factors associated with these mental health issues.

## Materials and methods

The present study was a community-based cross-sectional study conducted over one year among urban slum women in Kolar, from August 2023 to 2024. The study was carried out in a single urban slum with a population of 6,200. The study population consisted of urban slum women in their perimenopausal age. The sample size calculation was based on a meta-analysis that reported a prevalence of depression in perimenopausal and postmenopausal women in India at 42.47% [12]. With an error margin of 10% and a 95% confidence level, the sample size was estimated using the formula n = z2pq/d2, where prevalence (p) was 42.47%, and the absolute error (d) was 10%. The calculated sample size was 96, which, considering a cluster effect of 1.5%, was rounded up to 148.

A line listing of perimenopausal women aged 40-50 years was conducted. The study used the Stages of Reproductive Aging Workshop (STRAW) criteria to categorize perimenopausal women. According to the STRAW criteria, menopause is considered central to the staging system and labeled as point zero (0). The stages preceding the FMP are −5 to −1, and the stages following it are +1 to +2. Stages −5 to −3 encompass the reproductive interval; −2 to −1 reflect the menopausal transition; and +1 to +2 define postmenopause. For the present study, perimenopausal women were defined as those in stages −2 to +1a of the STRAW criteria. This stage is further divided into early and late phases. The early phase of perimenopause is characterized by a variable cycle length of more than seven days, and the late phase involves two or more skipped cycles with 60 or more days of amenorrhea. Follicle-stimulating hormone (FSH) levels are typically high in these phases [13].

Perimenopausal women aged 40-50 years residing in selected slums were included in the study, while those diagnosed with mental illness or chronic conditions such as tuberculosis, HIV, or those who were chronically bedridden and could influence mental health, were excluded. A preliminary survey was conducted to identify eligible participants fitting the inclusion and exclusion criteria. Using simple random sampling, a sample of 148 women was selected for the study. A total of 188 households with perimenopausal women were numbered from 1 to 188, and 148 were randomly selected through a lottery method.

A pretested structured questionnaire was used to collect sociodemographic details. To assess mental health status, the Depression Anxiety Stress Scale-21 (DASS-21) was used. The DASS-21 is a shorter version of the original DASS, consisting of 21 items that assess the severity of symptoms related to depression, anxiety, and stress. Respondents rate each item on a 4-point Likert scale from 0 (did not apply to me at all) to 3 (applied to me very much). Scores are then calculated for each scale, with higher scores indicating more severe symptoms. The scoring system is as follows: Depression: 0-9 (normal), 10-13 (mild), 14-20 (moderate), 21-27 (severe), and 28+ (extremely severe) for depression-specific questions (3, 5, 10, 13, 16, 17, 21). Anxiety: 0-7 (normal), 8-9 (mild), 10-14 (moderate), 15-19 (severe), and 20+ (extremely severe) for anxiety-specific questions (2, 4, 7, 9, 15, 19, 20). Stress: 0-14 (normal), 15-18 (mild), 19-25 (moderate), 26-33 (severe), and 34+ (extremely severe) for stress-specific questions (1, 6, 8, 11, 12, 14, 18) [14]. Data were collected using an interview technique, which lasted no longer than 15 minutes, by a coinvestigator who had prior training in using the DASS-21 tool.

Ethical consideration

Informed written consent was obtained from all participants before data collection. Ethical clearance for the study was granted by the Central Ethics Committee of Sri Devaraj Urs Academy of Higher Education and Research on September 14, 2024 (SDUAHER/KLR/R&D/CEC/S/PG/52/2024-25). The autonomy and confidentiality of the study participants were upheld throughout the research, and no participants were harmed during the course of the study.

Statistical analysis

All data were entered into a Microsoft Excel spreadsheet (Windows version; Microsoft Corporation, Redmond, WA, USA) and analyzed using IBM SPSS Statistics for Windows, Version 22.0 (Released 2013; IBM Corp., Armonk, NY, USA). Descriptive statistics were applied. To assess the association between factors and stress, anxiety, and depression, the Chi-square test was used, with a significance level set at a p-value of less than 0.05. Binary logistic regression analysis was performed to determine the ORs.

Operational definitions

Perimenopausal women were defined according to the STRAW criteria. Homemakers were participants who managed individual households and did not engage in paid work outside the home. Participants were categorized as single if they were either widowed or separated. Those with less than seven years of formal education were classified as having “no formal education.” Comorbidities were defined as participants diagnosed with either diabetes mellitus or hypertension at a medical center using appropriate diagnostic protocols. The Modified Bramha Govind (BG) Prasad 2024 Classification was used to stratify participants based on their family income and the number of family members [15].

**Figure 1:**
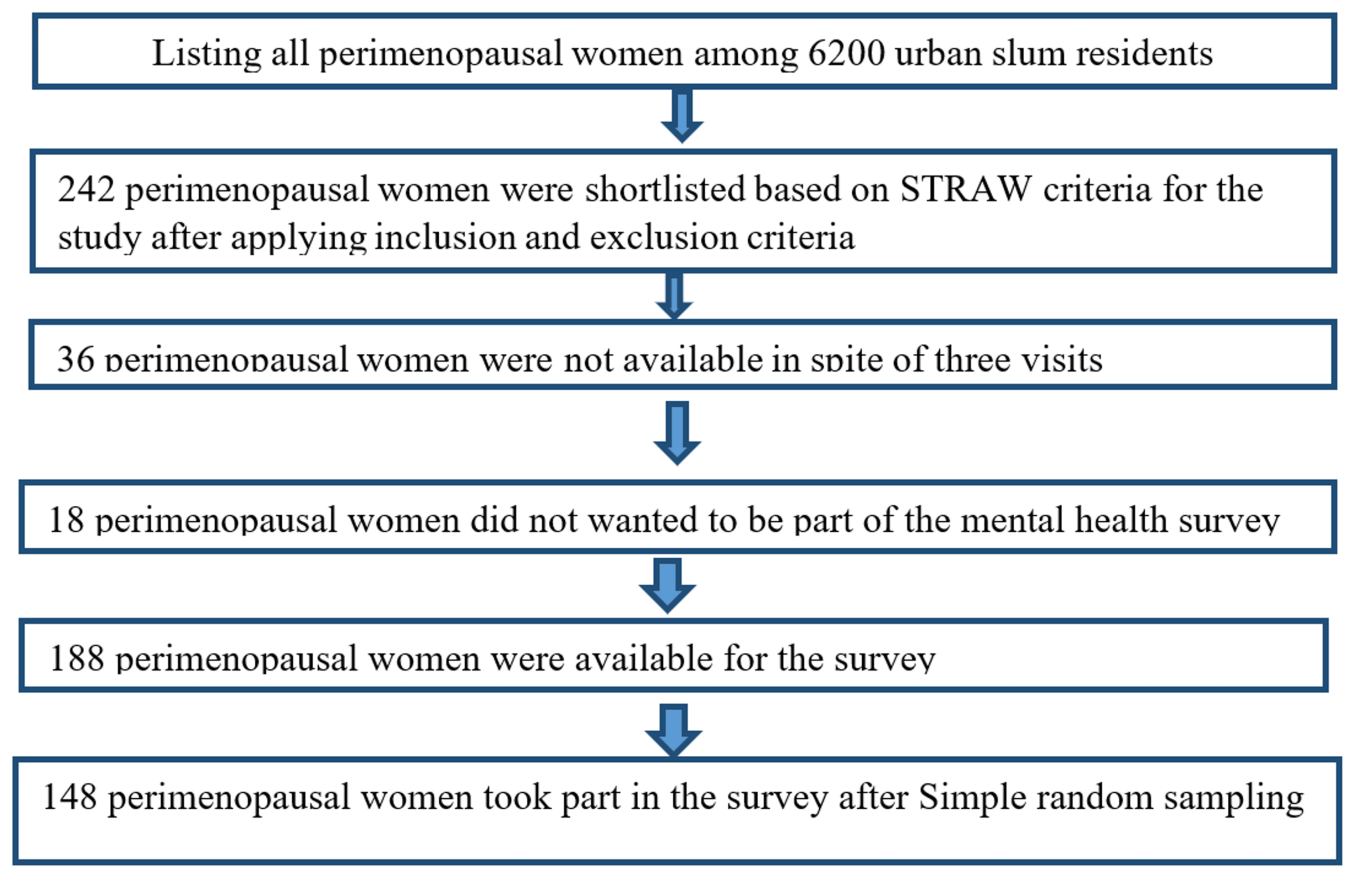
Recruitment and study procedure for perimenopausal women participants

Urban slums are characterized by inadequate housing, poor living conditions, lack of access to clean water, and overcrowding, all of which can negatively affect the health and well-being of residents. These areas often face various health challenges, including a higher prevalence of noncommunicable diseases, socioeconomic vulnerabilities, and limited access to routine screening and diagnostic healthcare [16]. Figure 1 presents a flowchart depicting the selection process of perimenopausal women participants from the chosen urban slum.

## Results

A total of 148 women from urban slums participated in the study, meeting the inclusion and exclusion criteria. Among them, 89 (60.1%) were homemakers, 85 (57.4%) had completed primary schooling, and 63 (42.6%) had comorbidities such as diabetes mellitus and hypertension. Additionally, 131 (88.5%) were currently married, and 130 (87.8%) had more than two children. The mean age of the perimenopausal women was 43.6 years, and the mean age of menarche was 12.4 years. All participants were classified as belonging to Class V in the Modified Bramha Govind (BG) Prasad 2024 Classification and held below poverty line cards (Table 1).

**Table 1:** Distribution of perimenopausal women according to sociodemographic factors

Variable	Category	Frequency (N)	Percentage (%)
Age in years	Less than 45 years	79	53.4
	More than 45 years	69	46.6
Occupation	Homemaker	89	60.1
	Work for wages	59	39.9
Education	No formal education	63	42.6
	Primary education	85	57.4
Comorbidities	Present	63	42.6
	Absent	85	57.4
Marital status	Married	131	88.5
	Single	17	11.5
Number of children	Two or fewer	18	12.2
	More than two	130	87.8

Among the 148 urban slum women who participated in the study, 124 (83.8%) reported moderate stress, 112 (75.7%) experienced moderate anxiety, and 82 (55.4%) exhibited severe depression, as assessed using the DASS-21 questionnaire (Table 2).

**Table 2:** Distribution of perimenopausal women according to stress, anxiety, and depression status DASS-21, Depression Anxiety Stress Scale-21

DASS		Frequency (N)	Percentage (%)
Stress	Mild	24	16.2
	Moderate	124	83.8
Anxiety	Mild	36	24.3
	Moderate	112	75.7
Depression	Moderate	66	44.6
	Severe	82	55.4

The present study found a significant association between factors such as educational status and marital status with stress and depression. Among the illiterate participants, 57 (90.5%) experienced stress, and this association between stress and education status was statistically significant (p < 0.05). Additionally, 79 (60.3%) of the married participants had depression, and this association between depression and marital status was also statistically significant (p < 0.05) (Table 3).

**Table 3:** Association between various factors in perimenopausal women with stress, anxiety, and depression status * The level of significance was found to be less than 0.05.

Variable	Category	Stress		Anxiety		Depression	
		Mild	Moderate	Mild	Moderate	Moderate	Severe
Age in years	Less than 45 years	14 (17.7%)	65 (82.3%)	21 (26.6%)	58 (73.4%)	28 (35.4%)	51 (64.6%)
	More than 45 years	10 (14.5%)	59 (85.5%)	15 (21.7%)	54 (78.3%)	38 (55.1%)	31 (44.9%)
	Chi-square value	2.2		1.2		5.7	
	p-value	0.2		0.3		0.01^*^	
Education	Illiterate	6 (9.5%)	57 (90.5%)	13 (20.6%)	50 (79.4%)	28 (44.4%)	35 (55.6%)
	Primary education	18 (21.2%)	67 (78.8%)	23 (27.1%)	62 (72.9%)	38 (44.7%)	47 (55.3%)
	Chi-square value	3.6		1.4		1.2	
	p-value	0.04^*^		0.2		0.5	
Children	Less than 2	5 (27.8%)	13 (72.2%)	6 (33.3%)	12 (66.7%)	9 (50.0%)	9 (50.0%)
	More than 2	19 (14.6%)	111(85.4%)	30 (23.1%)	100 (76.9%)	57 (43.8%)	73 (56.2%)
	Chi-square value	2.6		1.9		1.8	
	p-value	0.5		0.1		0.23	
Occupation	Homemaker	13 (14.6%)	76 (85.4%)	21 (23.6%)	68 (76.4%)	38 (42.7%)	51 (57.3%)
	Works for wages	11 (18.6%)	48 (81.4%)	15 (25.4%)	44 (74.6%)	28 (47.5%)	31 (52.5%)
	Chi-square value	2.4		1.2		3.2	
	p-value	0.3		0.47		0.34	
Comorbidities	Yes	14 (22.2%)	49 (77.8%)	16 (25.4%)	47 (74.6%)	32 (50.8%)	31(49.2%)
	No	10 (11.8%)	75 (88.2%)	20 (23.5%)	65 (76.5%)	34 (40.0%)	51 (60.0%)
	Chi-square value	2.9		1.2		1.7	
	p-value	0.4		0.3		0.2	
Marital status	Married	19 (14.5%)	112 (85.5%)	31 (23.7%)	100 (76.3%)	52 (39.7%)	79(60.3%)
	Others	5 (29.4%)	12 (70.6%)	5 (29.4%)	12 (70.6%)	14 (82.4%)	3 (17.6%)
	Chi-square value	2.4		1.9		11.8	
	p-value	0.4		0.2		0.01^*^	

Binary logistic regression analysis was performed to examine the association between various factors and stress, using women with mild stress as the reference category for comparison. The analysis revealed that perimenopausal women with no formal education (adjusted OR: 3.3, p = 0.03) and homemakers (adjusted OR: 1.4, p = 0.02) were significantly associated with moderate stress, showing higher ORs with statistically significant p-values (Table 4).

**Table 4:** Binary logistic regression analysis of various factors with stress A p-value less than 0.05 is considered statistically significant.

Factor	B	SE	p-value	OR	95% CI for EXP(B)	
					Lower	Upper
No formal education	1.206	0.571	0.035	3.339	1.09	10.229
Number of children (two or less)	-1.16	0.658	0.078	0.314	0.086	1.138
Age less than 45 years	-0.062	0.503	0.902	0.94	0.351	2.519
Homemakers	0.356	0.484	0.029	1.428	0.553	3.688
Presence of Comorbidities	-1.087	0.497	0.462	0.337	0.127	0.894
Married	1.043	0.648	0.107	2.838	0.797	10.108
Constant	0.876	0.769	0.255	2.400		

Binary logistic regression analysis was performed to examine the association between various factors and anxiety, using women with mild anxiety as the reference category for comparison. The analysis showed that perimenopausal women with no formal education (adjusted OR: 1.4, p = 0.4), homemakers (adjusted OR: 1.1, p = 0.8), and married women (adjusted OR: 1.3, p = 0.5) had higher ORs for experiencing moderate anxiety. However, none of these associations were statistically significant (Table 5).

**Table 5:** Binary logistic regression analysis of various factors in perimenopausal women with anxiety A p-value less than 0.05 is considered statistically significant.

Factor	B	SE	p-value	OR	95% CI for EXP(B)	
					Lower	Upper
No formal education	0.351	0.426	0.41	1.421	0.616	3.277
Number of children (two or less)	0.579	0.564	0.304	0.56	0.186	1.692
Age less than 45 years	-0.209	0.419	0.617	0.811	0.357	1.842
Homemakers	0.096	0.398	0.809	1.101	0.504	2.404
Presence of Comorbidities	-0.201	0.399	0.614	0.818	0.374	1.789
Married	0.326	0.593	0.582	1.386	0.433	4.43
Constant	0.931	0.685	0.174	2.536		

Binary logistic regression analysis was conducted to examine the association between various factors and depression, using women with moderate depression as the reference category for comparison. The analysis revealed that perimenopausal women with no formal education (adjusted OR: 1.4, p = 0.3), those aged less than 45 years (adjusted OR: 2.1, p = 0.04), homemakers (adjusted OR: 1.3, p = 0.4), and married women (adjusted OR: 6.3, p < 0.01) all had higher ORs for experiencing severe depression. However, the associations for marital status and age less than 45 years were statistically significant, with p-values less than 0.05 (Table 6).

**Table 6:** Binary logistic regression analysis of various factors in perimenopausal women with depression A p-value less than 0.05 is considered statistically significant.

Factor	B	SE	p-value	OR	95% CI for EXP(B)	
					Lower	Upper
No formal education	0.393	0.389	0.312	1.482	0.692	3.174
Number of children (two or less)	-0.196	0.565	0.729	0.822	0.272	2.489
Age less than 45 years	0.779	0.383	0.042	2.179	1.029	4.615
Homemakers	0.283	0.363	0.437	1.327	0.651	2.704
Presence of Comorbidities	-0.527	0.365	0.149	0.59	0.289	1.208
Married	1.847	0.675	0.006	6.338	1.689	23.781
Constant	-1.93	0.757	0.011	0.145		

## Discussion

The present study was a cross-sectional investigation conducted over one year in an urban slum, focusing on perimenopausal women aged 40-50 years who met the STRAW criteria. A total of 148 urban slum perimenopausal women participated in the study. Among these participants, 89 (60.1%) were homemakers, 85 (57.4%) had completed primary schooling, and 63 (42.6%) had comorbidities. The majority were married and had more than two children. The mean age of the participants was 43.6 years, with a mean age of menarche of 12.4 years. Regarding mental health, 124 (83.8%) exhibited moderate stress, 112 (75.7%) experienced moderate anxiety, and 82 (55.4%) had severe depression.

Global studies on perimenopausal women have reported similar prevalences of depression and anxiety. For example, a study in Brazil, using the Mini-International Neuropsychiatric Interview (MINI) tool, found anxiety and depression in 58% and 62% of perimenopausal women, respectively. This study also employed the STRAW criteria [17]. In China, a cohort study of 1,216 community-dwelling women showed that 39.5% reported depression during perimenopause, with comorbidities such as diabetes, hypertension, or hypothyroidism being risk factors for depression [18]. Another study by Saleh et al., involving 149 perimenopausal women, found 61.7% had anxiety and 26.8% had depression. Increased anxiety and depression scores were associated with worsened menopausal symptoms, sleep disturbances, and impact on daily routines [19]. Studies suggest that preexisting mental illness can be exacerbated during menopause. A study in Croatia found that anxiety-sensitive women were more vulnerable to perimenopausal distress due to reduced mental capacity, with greater psychological concerns expressed by those over 40 years of age [20]. In Australia, research indicated that perimenopause was linked to an increased risk of depression (incidence rate ratio (IRR) = 1.35, p = 0.008) and anxiety (IRR = 1.22, p = 0.030) in women without prior major depressive disorder (MDD) or generalized anxiety disorder [21]. A Swiss study highlighted factors like a history of depression and perceived stress as predictors of depression during perimenopause [22].

Medical conditions such as comorbidities (e.g., diabetes and hypertension) can influence mental health among perimenopausal women. A study in Austria indicated that having diabetes was a stronger risk factor for MDD in women than men, particularly between the ages of 40 and 55 [23]. A seven-year cohort study in China found that 41.2% of participants with chronic diseases such as diabetes, hypertension, and arthritis developed depression, with metabolic disorders and diabetes being significant risk factors [24]. In Poland, a study found that perimenopausal women experienced a poor quality of life, although no significant relationship between depression and diabetes was established [25]. These studies demonstrate that perimenopause is associated with a higher frequency of depressive symptoms compared to postmenopause. However, the present study did not find a significant association between comorbidities and anxiety, depression, or stress.

A narrative review of factors influencing mental health during perimenopause suggests that hormonal imbalances and social factors, such as poor relationships or childhood maltreatment, may worsen stress and anxiety during this transition [26]. A qualitative study in Australia highlighted the significant mental health impact of anxiety and depression during perimenopause, noting that social stigma related to menstruation and cultural factors made it difficult for women to cope with the changing menstrual and mental health challenges [27].

The strengths of this study include the use of a validated questionnaire to assess mental health status and its unique focus on urban slum women, who were selected based on standard inclusion criteria (STRAW criteria). However, there are several limitations. No FSH estimation was done to confirm the perimenopausal status of the women; instead, their menstrual histories were used. The cross-sectional design prevents the establishment of a temporal association between mental illnesses like depression, anxiety, and stress, and the perimenopausal phase. Additionally, potential confounding variables such as cultural stigma, access to healthcare, and socioenvironmental factors were not assessed, which may have influenced both the prevalence and reporting of mental health issues. The study was conducted in a single urban slum with a small sample size, limiting the generalizability of the findings.

Based on the results, it is recommended to screen for mental health issues such as anxiety, depression, and stress among women in the perimenopausal age group at primary health centers or urban health clinics, particularly in slum areas. It is important to raise awareness through information, education, and communication about the potential for these mental health conditions to worsen during perimenopause, sometimes requiring psychiatric attention.

## Conclusions

The present study provides valuable insights into the mental health status of women living in urban slums. Despite ongoing efforts, the healthcare system has yet to fully reach these populations, and mental health remains largely neglected. The study found that the prevalence of anxiety, depression, and stress among perimenopausal women in urban slums is relatively high, with educational status, marital status, and age being significant factors associated with these conditions. Given that perimenopause is a period of vulnerability for women, it is crucial to pay attention to the signs and symptoms of anxiety, depression, and stress to help women lead more productive lives.

Although various national programs aim to support women in urban slums, mental health continues to be overlooked. There is a need to raise awareness that the hormonal changes associated with menstruation cessation can contribute to mental health issues in perimenopausal women. It is essential to educate urban slum dwellers about mental health concerns and their potential solutions through targeted information, education, and communication activities.
